# Bi-directional prospective associations between objectively measured physical activity and fundamental motor skills in children: a two-year follow-up

**DOI:** 10.1186/s12966-019-0902-6

**Published:** 2020-01-02

**Authors:** Ada Kristine Ofrim Nilsen, Sigmund Alfred Anderssen, Kjersti Johannessen, Katrine Nyvoll Aadland, Einar Ylvisaaker, Jan Morten Loftesnes, Eivind Aadland

**Affiliations:** 1 Western Norway University of Applied Sciences, Faculty of Education, Arts and Sports, Institute of Sports, Food, and Natural Sciences, Campus Sogndal, Post box 133, 6851 Sogndal, Sogn og Fjordane Norway; 2 Department of Sports Medicine, Norwegian School of Sport Sciences, Post box 4014, Ullevål Stadion, 0806 Oslo, Norway

**Keywords:** Longitudinal association, Health behaviour, Motor competence, Motor development, Movement, Accelerometer, Reciprocal relationship, Preschool, Physical activity measurement

## Abstract

**Background:**

The direction of the longitudinal relationship between physical activity (PA) and fundamental motor skills (FMS) remains unclear. We evaluated the bi-directional, prospective relationships between intensity-specific physical activity (PA) and domain-specific fundamental motor skills (FMS) over 2 years in children attending preschool at baseline.

**Methods:**

A sample of 230 children (mean age at baseline 4.7 yr, 52% boys) from the 'Sogn og Fjordane Preschool Physical Activity Study' was measured 2 years apart. PA was assessed using ActiGraph accelerometers (GT3X+). FMS were evaluated by a test battery guided by the 'Test of Gross Motor Development 3' and the 'Preschooler Gross Motor Quality Scale'. PA outcomes were total PA (TPA [counts per minute]) and intensity specific PA and sedentary behaviour (SED) (min/day). FMS outcomes were locomotor, object control, and balance skills. Linear mixed model adjusting for potential co-variates was used to evaluate the bi-directional prospective associations between these variables, including the moderating effect of sex and age.

**Results:**

Baseline total PA, moderate-to-vigorous PA (MVPA), and vigorous PA predicted higher locomotor, object control, and balance skills at follow-up (standardized regression coefficient (β): 0.17 to 0.26, *p* = 0.002–0.017). Baseline SED predicted lower locomotor skills at follow-up (β: − 0.27, *p* = 0.012). Baseline light PA did not predict FMS at follow-up. Baseline FMS were not associated with PA or SED at follow-up.

**Conclusions:**

MVPA was positively associated with development of FMS in young children. In contrast, FMS were not related to future PA levels. Our results suggest promotion of MVPA is important for FMS development in young children.

## Background

Health related behaviours, such as physical activity (PA) and sedentary behaviour (SED), are typically established during early childhood, and evidence suggests that these behaviours track over time [1]. As PA levels are known to decrease by age in school-aged children and adolescents [2], the preschool years is a critical period to ensure sufficient levels of PA [3] for health and normal development [4–6]. Therefore, it is recommended that children engage in ≥ 60 min of moderate to vigorous PA (MVPA) daily [7]. However, many children fail to meet these guidelines [8–10].

In order to establish sufficient levels of PA during early childhood, research should aim to identify factors influencing PA behaviours [11]. Fundamental motor skills (FMS), including locomotor (moving the body through space, e.g., run, hop, jump), object control (manipulation and projecting of objects, e.g., catch or throw a ball), and balance skills (e.g., dynamic and static balance) [12], has been highlighted as important determinants of PA and other health related outcomes [13]. FMS’ are considered the 'building blocks' of more advanced, complex movements [14]. Children develop their FMS through engagement in PA [15], as increased PA provides more opportunities to promote neuromotor development, which in turn promotes FMS development [16–18]. At the same time, learning to move is a necessary skill underlying PA [18]. Proficiency in FMS is considered vital to achieve and maintain sufficient levels of PA [19, 20] and to develop more complex motor skills [13, 18]. Yet, many children have sub-optimal FMS levels [21–23].

Based on the conceptual model introduced by Stodden et al. in 2008 [18], the relationship between FMS and PA is likely to be bi-directional. In addition, the relationship may differ at different stages of a child’s development. While Stodden et al. hypothesised engagement in PA to be important for the development of FMS during the early years, FMS levels were hypothesised to become more important for PA participation as the child gets older (and becomes more motor competent) [18]. Numerous studies have examined the cross-sectional relationship between FMS and PA in children, supporting a low to moderate, positive association (*r* < 0.50) between FMS and levels of total PA (TPA), light PA (LPA), and MVPA [13, 19]. However, few longitudinal studies using objective measures of PA exist, and thus, the direction of the associations remains unclear.

A recent study by Schmutz et al. showed that FMS predicted higher accelerometer derived TPA and MVPA over a period of 12 months in children aged 2 to 6 years at baseline (*N* = 555) [24]. In addition, Venetsanou and Kambas [25] explored the longitudinal associations between FMS in preschoolers and PA measured with pedometers 10 years later (*N* = 106), and found that FMS during the preschool years predicted higher PA levels in adolescence. However, this study did not consider intensity-specific PA [25]. Importantly, though, these studies did not adjust for baseline PA levels, limiting their conclusions with regard to the direction of the association. Lopes et al., on the other hand, performed a longitudinal analysis showing that FMS positively predicted change in moderate PA (MPA), MVPA, and TPA in adolescents (*N* = 103) at 2-year follow-up [26]. Similarly, Larsen et al. found that motor performance positively predicted change in MVPA at 3-yr follow-up in their sample of 6–12 year old Danish children (*N* = 673) [27].

Since previous longitudinal studies primarily have focused on FMS as a determinant of PA, less is known about the predictive role of PA on FMS development. Although Barnett et al. found that MVPA at age 3.5 years was positively associated with locomotor skills at age 5 in a sample of preschoolers (*n* = 127) [28], their results are limited by the lack of adjustment for baseline levels of the outcome.

Only one previous study have investigated the bi-directional, prospective relationship between objectively measured PA and FMS in childhood. Lima et al. found that vigorous PA (VPA) and FMS presented a direct bi-directional, positive, prospective association over a 7-year follow-up of 513 children aged 6–13 years in the Copenhagen School Child Intervention Study (CoSCIS) [29]. Thus, their results correspond with the proposed model of Stodden et al. [18]. However, the authors urge future studies to investigate whether the strength of the associations between PA and FMS change during childhood [29]. In addition, Lima et al. only tested FMS within the locomotor domain; thus, more longitudinal research including other aspects of FMS (e.g., object control and balance skills) is needed.

To the best of our knowledge, no previous study has investigated the prospective, bi-directional relationship between PA and FMS in preschoolers using objective measures of PA. Considering the benefits of both PA and FMS for future health, an improved understanding of these variables’ interrelationships is an important public health focus in young children. Therefore, the aim of this study was to examine the prospective, bi-directional relationship between intensity-specific PA and domain-specific FMS in preschool-aged children over a period of 2 years.

## Methods

### Study design and recruitment of participants

The present study is a longitudinal analysis based on data from the 'Sogn og Fjordane Preschool Physical Activity Study' (PRESPAS) [30, 31]. PRESPAS was conducted in Sogn og Fjordane county, a rural area in western Norway, between September 2015 and June 2016 and involved in total 1308 children aged 2.7–6.5 years (born in 2010–2012) from 68 preschools (response rate 68%). The present study is based on a subsample of 376 invited children from 20 preschools, providing data at baseline (2015–2016) and at a two-year follow-up (September–October 2017).

Parents of all participating children received oral and written information about the study and provided written consent prior to testing. Preschools (at baseline and follow-up) and schools (at follow-up) received information about the study and agreed to participate in the study. We explained the procedures according to the children’s level of understanding. The Norwegian Centre for Research Data (NSD) approved the study (reference numbers: 39061 and 48016).

### Procedures

#### Physical activity measurement

PA was measured using the ActiGraph GT3X+ accelerometer (ActiGraph, LLC, Pensacola, Florida, USA) [32]. Children wore an elastic belt with the accelerometer on the right hip, and were instructed to wear the monitor at all times for 14 consecutive days, except during water-based activities and while sleeping (at night). Accelerometers were initialized with a sampling rate of 30 Hz and analysed using 1-s epochs using the KineSoft software (KineSoft version 3.3.80, Loughborough, UK). Periods of ≥20 min of zero counts were defined as non-wear time [33]. Our criterion for a valid day was ≥480 min of wear time accumulated between 06:00 and 24:00 h. We included all children who provided ≥4 days of valid PA data in the analysis. Children were asked to perform three PA-registration periods during the baseline year (autumn 2015, winter, and spring/summer 2016), and one PA-measurement at follow-up (autumn 2017), providing up to 6 weeks of PA data at baseline, and 2 weeks at follow-up. An average of the three PA measurements is used at baseline (in case of one missing observation, a mean of the two remaining PA registrations were used). PA outcomes were TPA (counts per minute [cpm]) and intensity-specific PA, reported as SED (≤ 100 cpm), LPA (LPA) (101–2295 cpm), MPA (2296–4011), VPA (≥4012 cpm), and MVPA (min/day) (≥2296 cpm), as proposed by Evenson et al. [34].

#### Fundamental motor skills

To measure FMS, we developed a test battery guided by the 'Test of Gross Motor Development 3' (TGMD-3) [35, 36]. TGMD-3 is designed for children aged 3–10 years, and originally based on observation of children’s movements across 13 tasks within the two domains: locomotion (run, skip, slide, gallop, hop, and horizontal jump) and ball/object control (hereafter referred to as 'object control') (overhand throw, underhand throw, catch, dribble, kick, one-hand strike, and two-hand strike). We modified this test battery to reduce the participant and researcher burden, and at the same time cover the three main domains of FMS by including balance skills [37, 38]. We therefore included six movement tasks from the TGMD-3 battery (run, horizontal jump, hop, catch, overhand throw, and kick), in addition to three movement tasks within the balance domain (single leg standing, walking line forward, and walking line backward) from the 'Preschooler Gross Motor Quality Scale' (PGMQ) proposed by Sun et al. [37], in our assessment of FMS. The specific skills were selected based on their relevance (e.g., some of the movement tasks in the TGMD-3, like the baseball strike and dribble, are less common and therefore less relevant in assessments of Norwegian children), and variety (e.g., including object control skills related to both hands and feet, and adding both static and dynamic balance tests) in terms of broadly capturing children’s skills within the three FMS domains.

FMS were measured one time at baseline (autumn 2015 - winter 2016), and one time at follow-up (autumn 2017). Children were evaluated in small groups (4–5 children) during preschool/school hours, and were asked to perform the nine movement tasks in a safe environment with enough space to move freely. Each child performed each skill twice and skills were completed in a standardised order, taking approximately 25–30 min per group. The test teams consisted of one instructor who provided a verbal description and demonstration of the required skill, while a separate rater observed and scored the performance. We administered the FMS measurements according to TGMD-3 (locomotor and object control skills) and PGMQ (balance skills) protocols. Children were scored quantitatively based on a qualitative evaluation of whether the child did or did not demonstrate specific process criteria for each skill/item based on the original scoring procedures for TGMD-3 (marked as either absent: “0” or present: “1”) [35–37]. The children had two trials per task, and the score from trial 1 and 2 were summed, thus - providing a score of 0 to 2 points per criteria. The criteria scores were summed for each item and each domain, providing domain scores of maximum 24 points for locomotor and balance skills (4 criteria per item, 3 items), and maximum 20 points for object control skills (3 criteria for 'catch' and 'kick', 4 criteria for 'overhand throw'). In total, six raters took part in the assessment of FMS. Prior to the data collection, all raters were thoroughly trained in how to instruct and score children in the different movement tasks. Inter-rater reliability (ICC) (based on in-field concurrent scoring of 26 children) was 0.90 for the locomotor items, 0.74 for the object control items, and 0.86 for the balance items.

#### Anthropometrics

We assessed children’s body weight and height during preschool hours. Body weight was measured to the nearest 0.1 kg using an electronic scale (Seca 899, SECA GmbH, Hamburg, Germany), and height was measured to the nearest 0.1 cm with a portable stadiometer (Seca 217, SECA GmbH, Hamburg, Germany). Body weight and height were measured at the same time as PA during baseline and follow-up (i.e., three times during the baseline year, and one time at follow-up). Body mass index (BMI, kg/m^2^) was calculated and used as a continuous variable in the association analyses (an average of the three baseline measurements is used). Children were additionally classified as normal weight, overweight, or obese based on criteria proposed by Cole et al. [39] for descriptive purposes.

#### Other covariates

Children’s sex, age, and parental socioeconomic status (SES, based on the highest education level and the highest yearly income of mother or father) were assessed using a questionnaire completed by each child’s mother and/or father at baseline. The included co-variates were chosen based on known influence on PA and FMS outcomes [2, 40, 41].

### Statistical analysis

**Fig. 1:**
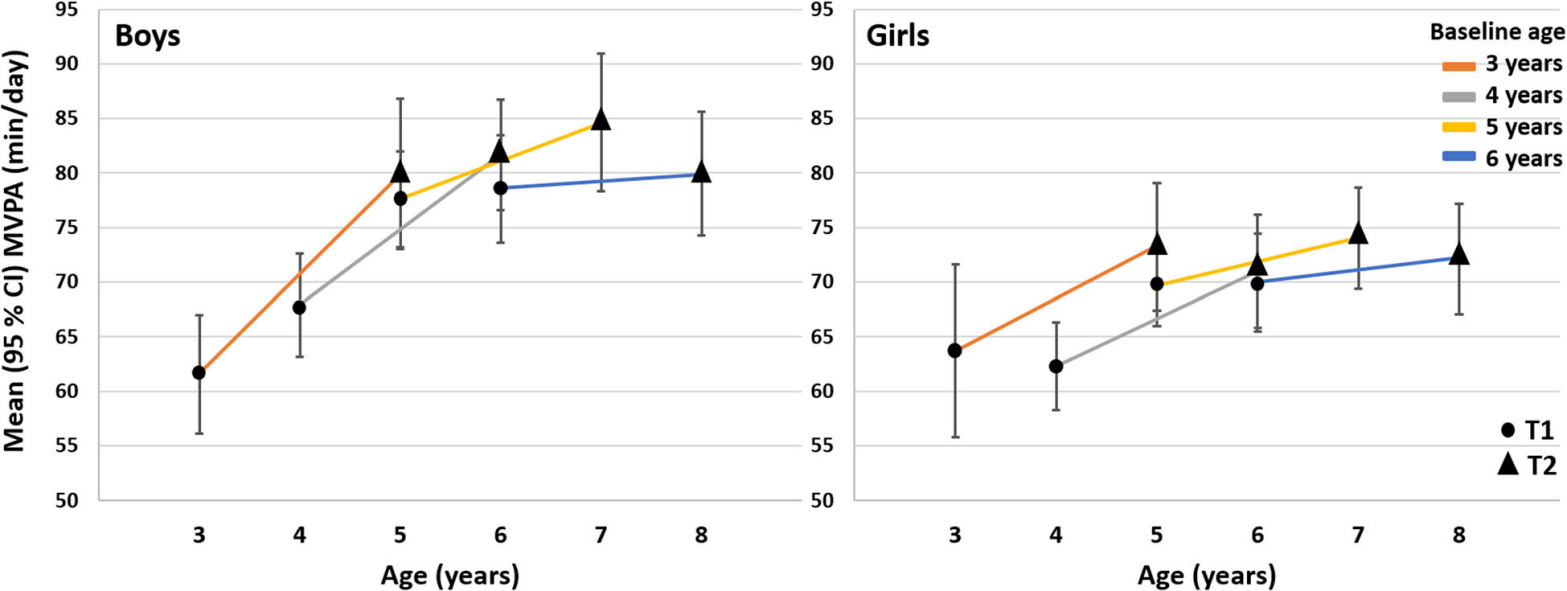
Development in moderate-to-vigorous physical activity (MVPA) from baseline (T1) to follow-up (T2) in boys and girls by age

Children’s characteristics, FMS, PA, and SED were reported as frequencies, means, and standard deviations (SD), except for the number of valid days of accelerometer data, which was reported as the median. We tested for differences in characteristics between children providing valid PA and FMS data at both time points and those who did not using a two-level linear mixed model for continuous outcomes and a generalized estimating equation using an exchangeable correlation structure for categorical outcomes, to account for clustering among preschools. We used Pearson’s correlations, change scores, and paired sample t-test to describe the differences in anthropometrics, FMS, and PA and SED between baseline and follow-up. Age-groups were based on median split (50% youngest, 50% oldest) for descriptive purposes in Additional file 3: Figure S1, and age-categories (≤ 3.49 years = 3; 3.50–4.49 years = 4; 4.50–5.49 years =5; ≥ 5.50 years = 6) for reporting of age-specific estimates in Fig. 1 and Additional file 1: Table S1 and Additional file 2: Table S2. For Additional file 6: Table S5, age-equivalents were categorized according to TGMD-3 (i.e., 3-month intervals) [36]. The cross-sectional analyses of the relationship between PA and FMS at baseline and at follow-up (Additional file 5: Table S3 and Additional file 4: Table S4) were performed using a linear regression model adjusted for potential co-variates (same as reported below).

The prospective association analyses were performed using a two-level linear mixed model including clustering of observations within individuals. The outcome at follow-up (PA or FMS) was the dependent variable in all models, while the independent variables were PA or FMS at baseline and the following covariates: sex, baseline age, baseline BMI, parental education and income level, accelerometer wear time at both time points (when PA was the outcome) and the person scoring FMS at both time points (when FMS was the outcome). All prospective analyses were adjusted for baseline value of the outcome. The analyses was repeated using different PA variables (LPA, MPA, VPA, MVPA, TPA), SED, as well as FMS (locomotor, object control, and balance skills) as predictors and outcomes. We did sensitivity analysis including random intercepts for clusters (preschool or school) at follow-up (i.e., a three-level model). However, because results from the three-level and two-level models were similar, we only reported results from the two-level models.

Furthermore, we tested for interactions by sex (baseline exposure (PA or FMS) × sex) and age (baseline exposure (PA or FMS) × baseline age) by adding these interaction terms to the models described above. In all models, FMS, SED, and PA variables were analysed one by one to avoid multi-collinearity.

For reporting of prospective associations, all FMS and PA variables were standardized to z-scores for ease of interpretation, thus, the regression coefficients are given in SD units. All analyses were performed using IBM SPSS v. 24 (IBM SPSS Statistics for Windows, Armonk, NY; IBM Corp., USA). *p* < 0.05 indicated statistically significant findings.

## Results

### Descriptives

Children’s characteristics are presented in Table 1. All of the 376 invited children participated in at least one measurement of PA at baseline, whereas 238 (63%) and 257 (68%) children had valid FMS and PA data, respectively, at both baseline and follow-up (*n* = 292 children (78%) had valid PA data at all three time points during the baseline measurements). In total, 230 (61%) children provided valid PA and FMS data at both baseline and follow-up and were included in the analyses. Compared to the included children, excluded children (*n* = 146) had parents with lower education and income levels (*p* < 0.05), but were otherwise similar to the study sample.

**Table 1:** Children’s characteristics at baseline and follow-up

	n	Baseline 2015/2016	Follow-up 2017	Pearson’s correlations	Change	*P* values ^d^
Age (years)	*376*	4.7 (0.9)	6.4 (0.9)	*–*	*–*	*–*
Boys (%)	*376*	52%	–	*–*	*–*	*–*
Anthropometrics	*249*					
Body height (cm)		109 (7.8)	121 (7.6)	0.962, *p < 0.001*	12.0 (2.1)	*p < 0.001*
Body mass (kg)		19.0 (3.2)	23.6 (4.3)	0.912, *p < 0.001*	4.6 (1.9)	*p < 0.001*
BMI (kg x m^2^)		16.1 (1.3)	16.0 (1.7)	0.790, *p < 0.001*	−0.1 (1.0)	*p = 0.328*
Weight status^a^ (%)						
*Normal weight*		84%	85%	–	–	*–*
*Overweight*		15%	12%	–	–	*–*
*Obese*		1%	3%	–	–	*–*
Parental education level^b^	*326*					
*Upper secondary school (%)*		16%	–	–	–	–
*University < 4 years (%)*		29%	–	–	–	–
*University* *>* *4 years (%)*		55%	–	–	–	–
Fundamental motor skills^c^	*238*					
Locomotor skills		15.1 (4.4)	16.3 (4.0)	0.439, *p < 0.001*	1.2 (4.5)	*p < 0.001*
Object control skills		10.4 (2.9)	16.8 (2.9)	0.503, *p < 0.001*	6.4 (2.9)	*p < 0.001*
Balance skills		16.5 (4.9)	21.1 (3.4)	0.557, *p < 0.001*	4.6 (4.2)	*p < 0.001*
Physical activity	*257*					
Wear time (min/day)		692 (43)	724 (54)	0.495, *p < 0.001*	32 (50)	*p < 0.001*
SED (min/day)		474 (39)	503 (47)	0.616, *p < 0.001*	29 (38)	*p < 0.001*
TPA ([cpm])		722 (147)	741 (165)	0.522, *p < 0.001*	19 (154)	*p = 0.042*
LPA (min/day)		144 (16)	139 (18)	0.635, *p < 0.001*	−5 (15)	*p < 0.001*
MPA (min/day)		36 (6)	39 (8)	0.601, *p < 0.001*	3 (6)	*p < 0.001*
VPA (min/day)		34 (9)	39 (10)	0.580, *p < 0.001*	5 (9)	*p < 0.001*
MVPA (min/day)		70 (14)	77 (16)	0.610, *p < 0.001*	7 (14)	*p < 0.001*

All values are reported as means (standard deviations) unless stated otherwise. ^a^Weight status according to Cole et al., 2000. ^b^Parental education and income level: *highest level of mother or father used.* ^c^ Score range locomotor and balance skills: 0–24; object control skills: 0–20. ^d^ The change from baseline to follow-up was analysed with the use of a paired-sample T-test. *P*-values is statistic significant to the level of *p* < 0.05. *BMI* Body mass index, *SED* Sedentary time, *TPA* Total physical activity, *cpm* Counts per minute, *LPA* Light physical activity, *MPA* Moderate physical activity, *VPA* Vigorous physical activity, *MVPA* Moderate-to-vigorous physical activity

### Development in PA and FMS

The children had a median of 12 valid days of PA at both baseline and follow-up. Both PA and FMS levels changed significantly over 2 years (Table 1). Results show greater increase in TPA, MPA, VPA, and MVPA from baseline to follow-up in boys compared to girls (Additional file 1: Table S1; Fig. 1 for MVPA). For SED, the trends were opposite (Additional file 1: Table S1). The development in PA was further strongly associated with age, with the younger children having a stronger, positive development in TPA, MPA, VPA, and MVPA (Additional file 1: Table S1 and Additional file 3: Figure S1; and Fig. 1 for MVPA), and a relatively smaller, positive development in SED, and smaller, negative development in LPA, when compared to the older children (Additional file 1: Table S1; Additional file 3: Figure S1).

Concerning FMS, skills within all three domains improved over 2 years (Table 1). There was no difference in FMS development between boys and girls; however, increased age at baseline were associated with greater development in object control skills (Additional file 2: Table S2).

### Cross-sectional relationships between PA and FMS

There were significant, positive associations between TPA, MPA, VPA, and MVPA and locomotor and object control skills at both time points (Additional file 5: Table S3 and Additional file 4: Table S4). SED was negatively associated with locomotor and object control skills at baseline, and with object control skills at follow-up. Balance skills were not associated with PA or SED, and LPA was not associated with FMS. Overall, the strength of the associations were similar at baseline and follow-up.

### Prospective, bi-directional relationships between PA and FMS

TPA, VPA, and MVPA at baseline predicted higher locomotor, object control, and balance skills at follow-up (*p* < 0.017) (Table 2). MPA predicted higher locomotor and balance skills at follow-up (*p* < 0.032). SED predicted lower locomotor skills (*p* = 0.012). LPA did not predict FMS at follow-up. We found no interactions with sex or age for the prospective relationship between PA (any intensity) or SED at baseline and FMS at follow-up (*p* = 0.122–0.995).

**Table 2:** Prospective associations between physical activity at baseline (exposure) and fundamental motor skills at follow-up (outcome) (*n* = 217)

		Outcome at follow-up		
		Locomotor skills	Object control skills	Balance skills
Exposure at baseline	TPA ([cpm])	0.23 (0.07, 0.39) ***p*** **= 0.006**	0.22 (0.07, 0.36) ***p*** **= 0.004**	0.17 (0.03, 0.30) ***p*** **= 0.014**
	SED	−0.27 (−0.47, −0.06) ***p*** **= 0.012**	−0.19 (−0.38, −0.01) *p* = 0.061	−0.14 (−0.32, 0.05) *p* = 0.155
	LPA	0.10 (−0.04, 0.24) *p* = 0.154	0.09 (− 0.04, 0.21) *p* = 0.192	0.09 (− 0.04, 0.21) *p* = 0.164
	MPA	0.22 (0.07, 0.37) ***p*** **= 0.005**	0.13 (−0.01, 0.27) *p* = 0.077	0.15 (0.03, 0.28) ***p*** **= 0.032**
	VPA	0.25 (0.08, 0.41) ***p*** **= 0.003**	0.19 (0.05, 0.34) ***p*** **= 0.010**	0.20 (0.06, 0.33) ***p*** **= 0.005**
	MVPA	0.26 (0.09, 0.42) ***p*** **= 0.002**	0.18 (0.03, 0.33) ***p*** **= 0.017**	0.19 (0.05, 0.33) ***p*** **= 0.007**

All values are standardized β coefficients (95% CI), analysed with a linear mixed model. The models are adjusted for sex, baseline age, baseline body mass index, parental education- and income level, FMS assessor at baseline and at follow-up, baseline accelerometer wear time, and baseline value of the outcome. *TPA* Total physical activity, *cpm* Counts per minute, *SED* Sedentary behaviour, *LPA* Light physical activity, *MPA* Moderate physical activity, *VPA* Vigorous physical activity, *MVPA* Moderate-to-vigorous physical activity, *FMS* Fundamental motor skills. *P*-value in bold is statistic significant to the level of *P* < 0.05

When FMS were modelled as the exposure and PA as the outcome, there was no prospective associations (Table 3). Furthermore, we found no interactions with sex or age for the prospective relationship between FMS at baseline and PA (any intensity) or SED at follow-up (*p* = 0.055–0.957).

**Table 3:** Prospective associations between fundamental motor skills at baseline (exposure) and physical activity at follow-up (outcome) (*n* = 224)

		Outcome at follow-up					
		TPA ([cpm])	SED	LPA	MPA	VPA	MVPA
Exposure at baseline	Locomotor skills	0.06 (−0.08, 0.19) *p* = 0.422	0.001 (− 0.07, 0.07) *p* = 0.989	−0.07 (− 0.18, 0.05) *p* = 0.239	−0.02 (− 0.15, 0.11) *p* = 0.734	0.06 (− 0.08, 0.20) *p* = 0.386	0.02 (− 0.11, 0.15) *p* = 0.706
	Object control skills	0.05 (− 0.08, 0.17) *p* = 0.472	0.01 (− 0.06, 0.07) *p* = 0.879	−0.08 (− 0.19, 0.03) *p* = 0.138	0.04 (− 0.09, 0.16) *p* = 0.557	0.06 (− 0.07, 0.18) *p* = 0.374	0.05 (− 0.07, 0.17) *p* = 0.385
	Balance skills	−0.06 (− 0.19, 0.07) *p* = 0.351	0.04 (− 0.04, 0.11) *p* = 0.348	−0.10 (− 0.21, 0.02) *p* = 0.114	−0.08 (− 0.21, 0.05) *p* = 0.214	−0.04 (− 0.17, 0.09) *p* = 0.532	−0.06 (− 0.19, 0.06) *p* = 0.308

All values are standardized β coefficients (95% CI), analysed with a linear mixed model. The models are adjusted for sex, baseline age, baseline body mass index, parental education- and income level, FMS assessor at baseline, accelerometer wear time at baseline and follow-up, and baseline value of the outcome. *TPA* Total physical activity, *cpm* Counts per minute, *SED* Sedentary behaviour, *LPA* Light physical activity, *MPA* Moderate physical activity, *VPA* Vigorous physical activity, *MVPA* Moderate-to-vigorous physical activity. *P*-value is statistic significant to the level of *P* < 0.05

## Discussion

This study extends the current evidence regarding the relationship between PA and FMS by examining the prospective, bi-directional associations between intensity-specific PA and domain-specific FMS in young children. While baseline PA of at least moderate intensity predicted higher FMS at follow-up, baseline FMS were not predictive of future PA levels.

We found that children who engaged in more MVPA and VPA during the preschool years performed better on FMS’s (all domains) 2 years later. These findings are consistent with the few previous studies that have examined prospective associations between PA and FMS in children [28, 29]. Similar to Lima et al., we found that associations were stronger for VPA than MPA [29]. Furthermore, LPA at baseline did not predict any FMS variable at follow-up. There was also a negative, prospective association between baseline SED and locomotor skills at follow-up. Based on our results, for each additional SD in MVPA (≈15 min), the locomotor skill score increased by 0.26 SD (≈15% increase). In comparison, Lima et al. found that locomotor skills increased by 0.14 SD for each additional SD in MVPA [29]. Because small improvements in FMS may enhance physically active play opportunities, and because others find a bi-directional relationship between PA and FMS in older children [29], we regard this increase meaningful for children’s development. Therefore, in line with Barnett et al. [28], our findings shows the importance of MVPA during the preschool years for FMS development.

Contrary to the findings above, we did not observe any prospective associations between FMS at baseline and PA at follow-up.

In line with the theory by Stodden et al., one would expect that motor competent children experience greater success and enjoyment during physically active play, and therefore would participate more in PA [18]. Thus, the lack of prospective associations between FMS (predictor) and MVPA (outcome) contrasts previous findings [24–27, 29]. A direct comparison of our results with previous studies is, however, difficult due to differences in follow-up duration, age of participants and different assessment methods for FMS.

We hypothesise that the null-findings regarding prospective relationships between FMS (predictor) and PA (outcome) could be explained by the great development in FMS that happens during the preschool and early school years [38]. Since FMS improves substantially over 2 years in young children [24, 28, 40], it may be reasonable to believe that the current motor skill level would be more strongly related to MVPA than the previous skill level. Our results from the cross-sectional analysis, showing comparable associations between PA and FMS at both baseline and follow-up, may support this theory, although the direction of the association cannot be determined from cross-sectional analyses. Nevertheless, our results are in contrast to those of Barnett et al., who found no cross-sectional relationship between MVPA and FMS in children at age five [28]. Barnett et al. did, however, find a prospective association between MVPA at age 3.5 and locomotor skills at age 5, which is consistent with our findings. Although Barnett et al., did not investigate the bi-directional relationship between these variables, our studies combined suggest that PA is more important for FMS development than FMS is for PA development in normally developing preschoolers.

Previous research has shown that a certain level of FMS is important for various health and learning outcomes [13, 42], and for participation in PA [24, 29, 40]. However, our findings support the hypothesis of Stodden et al., which suggests that the association between PA and FMS could be in the opposite direction for young children [18]. Our findings suggest prior time spent in MVPA is more important to the current level of FMS, than the prior motor skill level is for the current amount of MVPA when children are between the age of ≈5–8. Stodden and co-workers also hypothesised that the relationships between FMS and PA strengthen as children age and develop [18]. Therefore, we would expect several interactions of age for the prospective associations between PA and FMS. However, no such interactions were present in our material. It should be kept in mind, though, that the lack of interactions could result from the narrow age-span of the included children. Importantly, as previous evidence mainly is derived from older children, more longitudinal studies starting at an early age, and with longer follow-up duration, are needed to investigate the moderating effect of age on the bi-directional relationship between PA and FMS in children.

Limited research has targeted the development in PA during the years of preschool and early primary school. Some studies have shown an increase in TPA and MVPA by age [24, 28, 43, 44], while others have found substantial declines in TPA and MVPA over time [45]. Findings from the present study support those of others showing that PA increases by age in young children, however, in the present study, the development in PA were highly dependent on the children’s age and sex. The change in both TPA, MPA, VPA, and MVPA from baseline to follow-up were greater in boys compared to girls. Not only are boys in general more physically active than girls [2, 30, 40], but they also exhibit a greater increase in PA by age, as previously shown over a 10 month period for the current sample [30]. Furthermore, the younger children had considerably greater increase in PA of higher intensities over time when compared to the older children. Also, the older children had a greater positive development in SED when compared to the younger children. Moreover, when investigating development in PA by age we find almost no change in MVPA in the oldest children (≈8 years at follow-up) when compared to the other age groups (Fig. 1). These results are in line with previous research [2], showing a peak in PA levels around the age of 5–6. Such findings are not surprising as the decline in PA is likely related to the transition from preschool to primary school, which is related to both environmental, social, and behavioural changes, and thus different opportunities for PA [2].

As expected, FMS improved over 2 years. The younger children had a greater increase in object control skills than the older, which makes sense as such skills are more advanced than locomotor and balance skills [12] likely to improve at a later stage of development (i.e., normally by age). Whereas previous studies have suggested that boys develop certain FMS' earlier than girls [46, 47], we did not observe any sex differences in the development of FMS herein. As sex-differences in FMS are evident in older children [41], the preschool years could be seen as a window of opportunity in terms of promoting motor development in girls.

### Strengths and limitations

We regard the prospective study design including measurements of both PA and FMS at two time points, which allowed for bi-directional analyses of these variables’ reciprocal relationships, a major strength of the present study. Importantly, this protocol provide stronger prospective evidence than some previous studies that have not been able to adjust for baseline levels of the outcome [24, 25, 28]. Thus, our results allow for strong inference of causality, although confounding cannot be excluded. Still, we accounted for several potential covariates (sex, age, BMI, parental income and education level, accelerometer wear time, and rater for FMS testing) to limit confounding. The follow-up time of 2 years is relatively long when compared to the majority of previous studies, especially considering the children’s young age. Additionally, the multiple PA measurements at baseline, the long monitoring periods (14 days), and the high compliance to the accelerometer protocol provides a solid foundation for investigating the focused relationships.

However, our results should be interpreted with some limitations in mind. It is possible that the null finding in terms of FMS being a predictor of future PA could be influenced by the difference in measurement error and, thus, sensitivity in measurement methods. Because the PA assessment at baseline consisted of up to 6 weeks of objective PA registration, which possibly provided a more precise estimate of PA compared to the single FMS assessment (at both time points), the results could be subject to differing measurement error and differing regression dilution bias [48]. When the more imprecise variable is modelled as the outcome the magnitude of effect is estimated accurately, but with wider confidence intervals [48]. In contrast, when the more imprecise variable is modelled as the exposure it tends to attenuate the regression coefficient [48]. In addition, FMS is a set of 'building blocks' of more advanced complex movements that is conceptualised, operationalised, and measured in different ways across studies [14]. Thus, FMS is hard both to define and to measure accurately.

A general limitation with accelerometer data is that they do not provide a true measure of true SED time or very high PA intensities, nor a correct classification of intensity in certain activities (e.g., cycling, swimming) [49]. Moreover, reporting of raw acceleration, which was not done for the present study, could improve comparability with future studies. Our findings should therefore be interpreted with limited classification accuracy of PA intensity and posture allocation taken into account. Another important issue in accelerometer data reduction and scoring is the choice of epoch length. Because children’s natural PA pattern is rather sporadic, with bouts of PA generally lasting < 10 s [50–53], it has been concluded that studies should apply shorter epochs than the traditional 60-s epoch duration to capture PA correctly [49]. Therefore, we used 1-s epochs to avoid loss of information and misclassification of PA intensity in the present study.

There is no established 'gold standard' of assessment of FMS in children [54]. In the present study, we used a test battery inspired by the TGMD-3 [35] as the TGMD-battery is widely used in preschoolers [54, 55]. However, the TGMD was developed in the USA and contains particular movement tasks that are less culturally relevant in Norway (e.g., the baseball strike and bouncing ball). Furthermore, the test does not contain balance tasks. To be able to measure FMS in a large study sample (*N* = 1308 children in the main sample of PRESPAS) and at the same time cover the three recognised domains of FMS [37, 38], we choose to modify and extend the TGMD-3. Thus, our results are limited by a lack of comparability with other studies using the TGMD-3. Moreover, the balance items included from the PGMQ [37] are only validated for children aged 3–6 years, and therefore not suited for approximately half of our sample at follow-up (mean age: 6.4 (0.9) years). Although we acknowledge this limitation, the mean values and SDs (Table 1) indicates no clear ceiling effect at follow-up.

The average parental educational level among children included in the analyses was higher than among the excluded children, and our sample was highly homogenous in terms of ethnicity and environmental factors. Also, a considerable number of children (*n* = 159 and *n* = 152 for the prospective analyses presented in Table 2 and 3, respectively) were excluded from the main analyses because of missing data in either predictors, outcomes or co-variates at baseline or follow-up. However, differences between included and excluded children were minor at baseline. Furthermore, our sample consisted of healthy children without known disabilities that could affect PA levels or FMS performance. This mean that caution should be exercised in generalising the results to populations comprising ethnic minorities, children with developmental disorders, or populations with lower SES.

### Perspectives

Our results suggest that an increased focus on promotion of MVPA during the preschool years can improve development of FMS. Given the additional benefits of MVPA on physical health and cognitive and social development during the early years [5], promotion of MVPA should be a priority public health strategy in this age group – ideally implemented in preschool and school settings where a large number of children can be reached regardless of social background.

As previously reported for this study material [31], the preschool arena is important for children’s MVPA. However, findings indicate that this environment stimulates boys, older children, and highly active children more successfully in terms of higher MVPA levels during preschool hours [31]. Even though sex and age are not modifiable factors, it is important that PA programs and social and physical environments (which are modifiable factors) are designed to provide opportunities for all children to increase their MVPA.

## Conclusions

In conclusion, PA of moderate to vigorous intensity predicted development of FMS in young children. In contrast, FMS did not predict future PA levels. Furthermore, FMS and MVPA increased by age within this sample of preschoolers, however, the development in PA is highly dependent on children’s sex and age. Our results highlight the importance of promoting MVPA for FMS development in children during the preschool years.

## Supplementary information

**Additional file 1: Table S1.** Prospective associations between exposing factors (sex and age) at baseline and physical activity at follow-up.

**Additional file 2: Table S2.** Prospective associations between exposing factors (sex and age) at baseline and fundamental motor skills at follow-up.

**Additional file 3: Figure S1.** The development in physical activity and sedentary behaviour over two years by sex and age (median split) in children attending preschool at baseline. Figure A: change in sedentary behaviour (SED); Figure B: change in light physical activity (LPA); Figure C: change in moderate physical activity (MPA); Figure D: change in vigorous physical activity.

**Additional file 4: Table S4.** Cross-sectional associations (main effects) between PA and FMS at follow-up.

**Additional file 5: Table S3.** Cross-sectional associations (main effects) between PA and FMS at baseline.

**Additional file 6: Table S5.** Mean sum scores (95% CI) at baseline and at follow-up for the specific items included in the evaluation of fundamental motor skills according to children’s age.

## Data Availability

The datasets used and analysed for the current study are available from the corresponding author on reasonable request.

## Abbreviations

BMI: Body Mass Index
cpm: Counts per minute
FMS: Fundamental motor skills
ICC: Intra-class correlation
LPA: Light physical activity
Min: Minutes
MPA: Moderate physical activity
MVPA: Moderate-to-vigorous physical activity
PA: Physical activity
SD: Standard deviation
SED: Sedentary behaviour
SES: Socioeconomic status
TPA: Total physical activity
VPA: Vigorous physical activity
