# Supplementary material for: Bi-directional prospective associations between objectively measured physical activity and fundamental motor skills in children: a two-year follow-up
Source: Int J Behav Nutr Phys Act. 2020 Jan 2;17:1. doi: 10.1186/s12966-019-0902-6 (PMC6941400; doi:10.1186/s12966-019-0902-6)
Supplement: Supplementary file 1 — Additional file 1: Table S1. Prospective associations between exposing factors (sex and age) at baseline and physical activity at follow-up. [file 12966_2019_902_MOESM1_ESM.docx]

**Table S1** Prospective associations between exposing factors (sex and age) at baseline and physical activity at follow-up (n=234).

|  |  | **Outcome at follow-up** | | | | | |
| --- | --- | --- | --- | --- | --- | --- | --- |
|  |  | **TPA ([cpm])** | **SED** | **LPA** | **MPA** | **VPA** | **MVPA** |
| **Exposure at baseline** | **Sex** | **p=0.013** | **p=0.038** | p=0.108 | **p<0.001** | **p=0.008** | **p<0.001** |
|  | *Girls (ref.)* | -- | -- | -- | -- | -- | -- |
|  | *Boys* | 43.7 (9.3, 78.0) | -6.0 (-11.6, -0.3) | 2.7 (-0.6, 6.0) | 3.4 (1.8, 5.0) | 2.8 (0.7, 4.8) | 6.2 (3.0, 9.4) |
|  | **Baseline age** (years) | **p trend <0.001** | **p trend <0.001** | **p trend <0.001** | **p trend=0.003** | **p trend <0.001** | **p trend <0.001** |
|  | *6 years (ref.)* | -- | -- | -- | -- | -- | -- |
|  | *5 years* | 34.0 (-12.4, 80.4)  p=0.150 | -9.1 (-16.6, -1.6)  **p=0.017** | 1.3 (-3.2, 5.7)  p=0.566 | 1.8 (-0.3, 3.8)  p=0.097 | 1.8 (-1.0, 4.5)  p=0.208 | 3.5 (-0.7, 7.8)  p=0.105 |
|  | *4 years* | 52.5 (2.0, 103.0)  **p=0.042** | -12.1 (-20.0, -4.1)  **p=0.003** | 5.6 (0.8, 10.3)  **p=0.022** | 1.7 (-0.5, 3.9)  p=0.134 | 3.6 (0.5, 6.6)  **p=0.021** | 5.3 (0.6, 9.9)  **p=0.026** |
|  | *3 years* | 104.0 (44.1, 163.9)  **p<0.001** | -23.6 (-33.0, -14.1)  **p<0.001** | 8.4 (2.7, 14.0)  **p=0.004** | 3.5 (0.9, 6.2)  **p=0.010** | 6.9 (3.3, 10.5)  **p<0.001** | 10.4 (4.9, 15.9)  **p<0.001** |

Results from a linear mixed model. The models are adjusted for sex, baseline age, baseline body mass index, parental education- and income level, baseline- and follow-up accelerometer wear time, and baseline value of the outcome. Results are reported as beta coefficients/minutes per day (95 % CI) relative to reference value, or as change per year. TPA: total physical activity; cpm: counts per minute; SED: sedentary behaviour; LPA: light physical activity; MPA: moderate physical activity; VPA: vigorous physical activity; MVPA: moderate-to-vigorous physical activity. *P*-value in bold is statistic significant to the level of *P*<0.05.
