# Supplementary material for: Bi-directional prospective associations between objectively measured physical activity and fundamental motor skills in children: a two-year follow-up
Source: Int J Behav Nutr Phys Act. 2020 Jan 2;17:1. doi: 10.1186/s12966-019-0902-6 (PMC6941400; doi:10.1186/s12966-019-0902-6)
Supplement: Supplementary file 2 — Additional file 2: Table S2. Prospective associations between exposing factors (sex and age) at baseline and fundamental motor skills at follow-up. [file 12966_2019_902_MOESM2_ESM.docx]

**Table S2** Prospective associations between exposing factors (sex and age) at baseline and fundamental motor skills at follow-up (n=217).

|  |  |  |  |  |
| --- | --- | --- | --- | --- |
|  |  | **Outcome at follow-up** | | |
|  |  | **Locomotor** | **Object control** | **Balance** |
| **Exposure at baseline** | **Sex** | p=0.585 | p=0.354 | p=0.632 |
|  | *Girls (ref.)* | -- | -- | -- |
|  | *Boys* | 0.07 (-10.18, 0.32) | 0.11 (-0.13, 0.35) | -0.06 (-0.29, 0.17) |
|  | **Baseline age** (years) | p trend=0.282 | **p trend=0.001** | p trend=0.155 |
|  | *6 years (ref.)* | -- | -- | -- |
|  | *5 years* | -0.31 (-0.64, 0.02) p=0.062 | -0.26 (-0.56, 0.05) p=0.101 | -0.02 (-0.33, 0.28)  p=0.870 |
|  | *4 years* | -0.48 (-0.86, -0.11)  **p=0.012** | -0.36 (-0.70, 0.01) **p=0.041** | -0.08 (-0.43, 0.28) p=0.664 |
|  | *3 years* | -0.05 (-0.56, 0.45) p=0.842 | -0.88 (-1.31, -0.45) **p<0.001** | -0.48 (-0.94, -0.03) **p=0.036** |

Results from a linear mixed model. The models are adjusted for sex, baseline age, baseline body mass index, parental education- and income level, baseline- and follow-up FMS assessor, and baseline value of the outcome. Results are reported as standardized beta coefficients (95 % CI) relative to reference value, or as change per year. *P*-value in bold is statistic significant to the level of *P*<0.05.
