# Supplementary figures and images for: Bi-directional prospective associations between objectively measured physical activity and fundamental motor skills in children: a two-year follow-up
Source: Int J Behav Nutr Phys Act. 2020 Jan 2;17:1. doi: 10.1186/s12966-019-0902-6 (PMC6941400; doi:10.1186/s12966-019-0902-6)

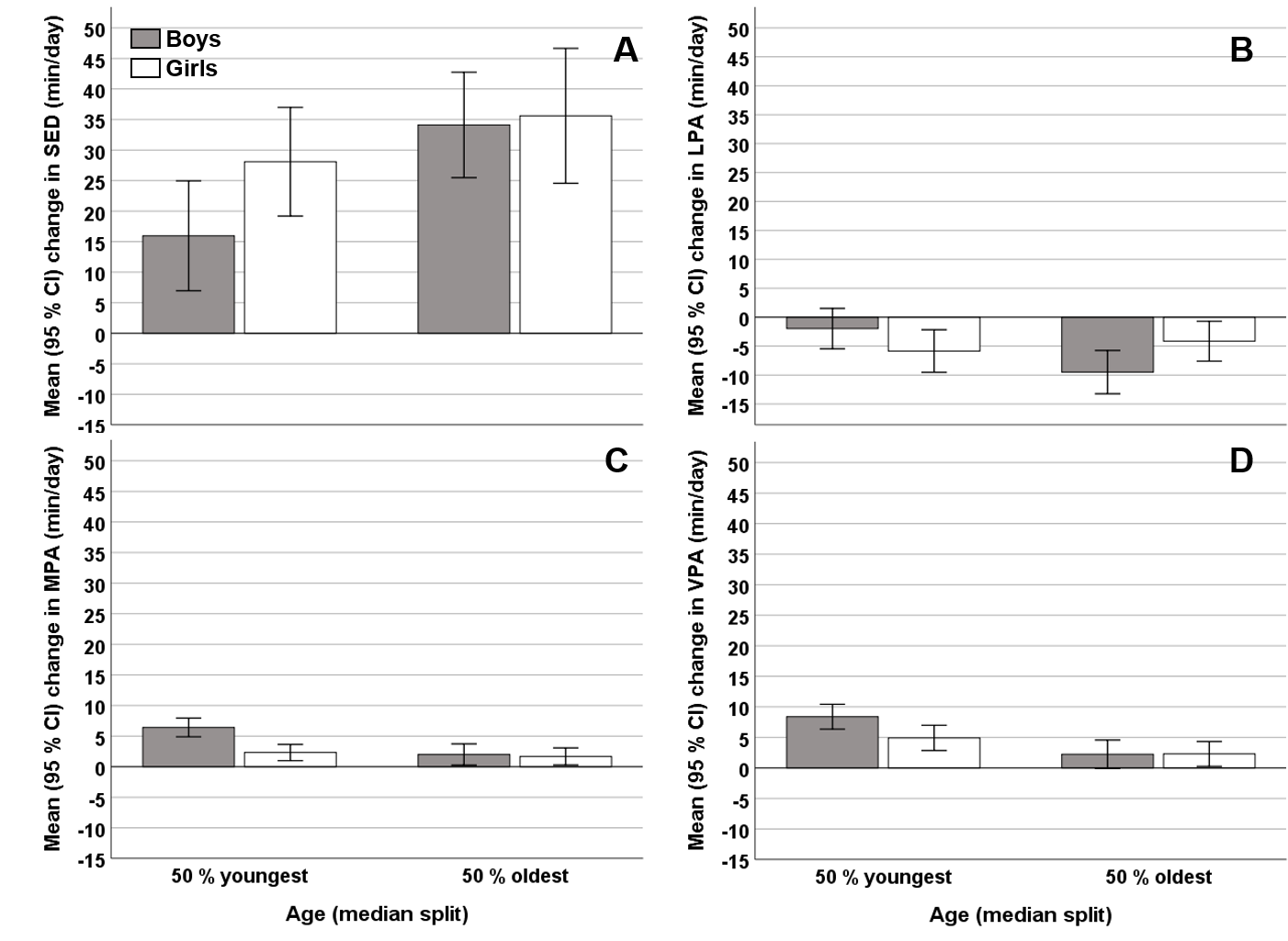

Supplement: Supplementary file 3 — Additional file 3: Figure S1. The development in physical activity and sedentary behaviour over two years by sex and age (median split) in children attending preschool at baseline. Figure A: change in sedentary behaviour (SED); Figure B: change in light physical activity (LPA); Figure C: change in moderate physical activity (MPA); Figure D: change in vigorous physical activity. [file 12966_2019_902_MOESM3_ESM.tif]
