# Supplementary material for: Bi-directional prospective associations between objectively measured physical activity and fundamental motor skills in children: a two-year follow-up
Source: Int J Behav Nutr Phys Act. 2020 Jan 2;17:1. doi: 10.1186/s12966-019-0902-6 (PMC6941400; doi:10.1186/s12966-019-0902-6)
Supplement: Supplementary file 4 — Additional file 4: Table S4. Cross-sectional associations (main effects) between PA and FMS at follow-up. [file 12966_2019_902_MOESM4_ESM.docx]

**Table S4:** Cross-sectional associations (main effects) between PA and FMS at follow-up (n=219)

|  | **Locomotor skills** | **Object control skills** | **Balance skills** |
| --- | --- | --- | --- |
| **TPA** | 0.30 (0.16, 0.43)** | 0.20 (0.07, 0.34)** | 0.11 (-0.02, 0.25) |
| **SED** | -0.20 (-0.42, 0.03) | -0.23 (-0.44, -0.01)* | -0.04 (-0.25, 0.18) |
| **LPA** | -0.06 (-0.20, 0.09) | 0.04 (-0.10, 0.17) | 0.02 (-0.12, 0.16) |
| **MPA** | 0.17 (0.03, 0.31)* | 0.15 (0.01, 0.28)* | 0.05 (-0.08, 0.19) |
| **VPA** | 0.31 (0.18, 0.44)** | 0.21 (0.08, 0.34)** | 0.10 (-0.03, 0.23) |
| **MVPA** | 0.28 (0.15, 0.42)** | 0.21(0.07, 0.34)** | 0.09 (-0.05, 0.23) |

Adjusted associations: sex, age, BMI, parental education- and income level, accelerometer wear time, test person FMS. Estimates are reported as standardized units (95 % CI). TPA: total physical activity; SED: sedentary behaviour; LPA: light physical activity; MPA: moderate physical activity; VPA: vigorous physical activity; MVPA: moderate to vigorous physical activity. ** p<0.01; *p<0.05.
