# Supplementary material for: Bi-directional prospective associations between objectively measured physical activity and fundamental motor skills in children: a two-year follow-up
Source: Int J Behav Nutr Phys Act. 2020 Jan 2;17:1. doi: 10.1186/s12966-019-0902-6 (PMC6941400; doi:10.1186/s12966-019-0902-6)
Supplement: Supplementary file 5 — Additional file 5: Table S3. Cross-sectional associations (main effects) between PA and FMS at baseline. [file 12966_2019_902_MOESM5_ESM.docx]

**Table S3:** Cross-sectional associations (main effects) between PA and FMS at baseline (n=303)

|  | **Locomotor skills** | **Object control skills** | **Balance skills** |
| --- | --- | --- | --- |
| **TPA** | 0.32 (0.22, 0.42)** | 0.20 (0.10, 0.31)** | 0.03 (-0.07, 0.13) |
| **SED** | -0.24 (-0.38, -0.09)** | -0.18 (0.34, 0.02)* | -0.02 (-0.16, 0.12) |
| **LPA** | 0.04 (-0.06, 0.14) | 0.05 (-0.05, 0.16) | -0.001 (-0.10, 0.09) |
| **MPA** | 0.26 (0.16, 0.37)** | 0.25 (0.14, 0.36)** | 0.030 (-0.07, 0.13) |
| **VPA** | 0.36 (0.26, 0.46)** | 0.24 (0.13, 0.35)** | 0.03 (-0.07, 0.13) |
| **MVPA** | 0.34 (0.24, 0.44)** | 0.26 (0.15, 0.37)** | 0.03 (-0.07, 0.14) |

Adjusted associations: sex, age, BMI, parental education- and income level, accelerometer wear time, test person FMS. Estimates are reported as standardized units (95 % CI). TPA: total physical activity; SED: sedentary behaviour; LPA: light physical activity; MPA: moderate physical activity; VPA: vigorous physical activity; MVPA: moderate to vigorous physical activity. ** p<0.01; *p<0.05.
