# Supplementary material for: Bi-directional prospective associations between objectively measured physical activity and fundamental motor skills in children: a two-year follow-up
Source: Int J Behav Nutr Phys Act. 2020 Jan 2;17:1. doi: 10.1186/s12966-019-0902-6 (PMC6941400; doi:10.1186/s12966-019-0902-6)
Supplement: Supplementary file 6 — Additional file 6: Table S5. Mean sum scores (95% CI) at baseline and at follow-up for the specific items included in the evaluation of fundamental motor skills according to children’s age. [file 12966_2019_902_MOESM6_ESM.docx]

**Table S5:** Mean sum scores (95 % CI) at baseline and at follow-up for the specific items included in the evaluation of fundamental motor skills according to children’s age (age equivalent, 3-month intervals)

| Test of Gross Motor Development 3 | | | | | | | | Preschooler Gross Motor Quality Scale | | |
| --- | --- | --- | --- | --- | --- | --- | --- | --- | --- | --- |
| Age equvalent | **n** | **Run**  (4 criteria) | **Horizontal jump**  (4 criteria) | **Hop**  (4 criteria) | **Two-hand catch**  (3 criteria) | **Overhand throw**  (4 criteria) | **Kick stationary ball**  (3 criteria*) | **Balance one foot**  (4 criteria) | **Walk line backward**  (4 criteria*) | **Walk line forward**  (4 criteria) |
| Baseline, n= 242 | | | | | | | | | | |
| 3-0 | 4 | 3.7 (2.2, 5.1) | 5.7 (2.2, 5.1) | 0.0 (0.0, 0.0) | 2.0 (2.0, 2.0) | 2.7 (0.2, 5.5) | 2.3 (0.9, 3.8) | 1.3 (-4.4, 7.1) | 3.3 (0.5, 6.2) | 3.7 (-1.5, 8.8) |
| 3-3 | 26 | 4.2 (3.5, 4.9) | 3.8 (3.0, 4.6) | 1.4 (0.5, 2.3) | 2.0 (1.7, 2.3) | 2.7 (2.0, 3.3) | 3.1 (2.4, 3.9) | 4.2 (3.2, 5.1) | 3.7 (3.0, 4.5) | 3.1 (2.4, 3.8) |
| 3-6 | 20 | 4.5 (3.6, 5.3) | 3.7 (2.8, 4.6) | 2.4 (1.1, 3.6) | 2.4 (1.9, 2.9) | 2.4 (1.7, 3.1) | 3.4 (2.8, 4.1) | 4.4 (3.1, 5.6) | 4.9 (4.0, 6.0) | 5.1 (3.2, 4.9) |
| 3-9 | 16 | 5.3 (4.5, 6.2) | 4.9 (3.8, 6.1) | 3.8 (2.5, 5.0) | 3.2 (2.6, 3.8) | 2.8 (2.1, 3.4) | 3.3 (2.6, 4.0) | 4.6 (3.4, 5.8) | 4.6 (3.6, 5.5) | 4.3 (3.3, 5.2) |
| 4-0 | 14 | 5.5 (4.8, 6.2) | 4.5 (3.4, 5.6) | 2.9 (1.8, 4.1) | 2.4 (1.9, 3.0) | 3.5 (2.8, 4.2) | 3.2 (2.6, 3.9) | 5.5 (4.1, 6.9) | 5.1 (4.4, 5.8) | 4.4 (3.7, 5.1) |
| 4-3 | 17 | 5.4 (4.6, 6.1) | 5.8 (4.9, 6.6) | 4.6 (3.2, 6.1) | 3.5 (2.7, 4.2) | 3.1 (2.5, 3.7) | 3.7 (2.8, 4.6) | 6.2 (5.3, 7.1) | 5.5 (5.0, 6.1) | 4.6 (4.0, 5.2) |
| 4-6 | 16 | 6.1 (5.7, 6.6) | 5.5 (4.5, 6,5) | 5.1 (4.5, 5.8) | 3.3 (2.5, 4.1) | 3.6 (3.2, 4.1) | 3.3 (2.6, 4.0) | 5.4 (4.8, 6.1) | 5.6 (5.8, 6.5) | 4.8 (3.7, 5.9) |
| 4-9 | 22 | 6.0 (5.6, 6.3) | 6.0 (5.2, 6.7) | 5.5 (4.7, 6.3) | 3.5 (2.9, 4.1) | 3.3 (2.7, 3.9) | 3.8 (3.2, 4.4) | 6.6 (6.0, 7.1) | 5.7 (5.2, 6.3) | 5.3 (4.6, 6.1) |
| 5-0 | 24 | 5.9 (5.3, 6.5) | 5.1 (4.1, 6.0) | 4.7 (3.7, 5.6) | 3.6 (3.0, 4.2) | 3.3 (2.7, 3.8) | 4.3 (3.7, 4.8) | 5.9 (4.7, 6.1) | 5.5 (4.6, 6.4) | 5.9 (5.2, 6.6) |
| 5-3 | 20 | 5.9 (5.3, 6.5) | 5.3 (4.4, 6.1) | 5.9 (5.2, 6.7) | 4.1 (3.4, 4.8) | 3.3 (2.7, 3.9) | 4.2 (3.4, 4.9) | 6.3 (5.4, 7.1) | 6.4 (5.8, 7.1) | 6.3 (5.6, 7.1) |
| 5-6 | 28 | 6.5 (5.9, 7.1) | 5.5 (4.9, 6.2) | 5.5 (4.7, 6.3) | 3.6 (3.0, 4.3) | 3.2 (2.5, 3.8) | 4.5 (4.0, 5.0) | 6.9 (6.3, 7.5) | 6.4 (6.0, 6.8) | 6.6 (6.0, 7.1) |
| 5-9 | 27 | 5.6 (5.1, 6.1) | 6.0 (5.4, 6.6) | 5.7 (5.1, 6.3) | 4.1 (3.5, 4.6) | 3.7 (3.1, 4.2) | 4.3 (3.7, 4.8) | 6.9 (6.4, 7.4) | 6.6 (6.0, 7.1) | 6.6 (5.9, 7.3) |
| 6-0 | 8 | 7.4 (6.6, 8.1) | 5.8 (4.2, 7.3) | 5.8 (3.8, 7.7) | 4.6 (3.4, 5.8) | 4.0 (2.7, 5.3) | 4.1 (3.4, 4.8) | 6.8 (5.4, 8.1) | 6.8 (5.4, 8.1) | 6.4 (5.0, 7.8) |
| Follow-up, n= 242 | | | | | | | | | | |
| 4-9 | 15 | 6.1 (5.1 7.0) | 4.5 (3.9, 5.2) | 4.1 (3.2, 5.0) | 4.8 (3.9, 5.7) | 5.4 (4.4, 6.4) | 4.3 (3.5, 5.2) | 5.3 (3.6, 6.9) | 6.1 (5.3, 7.0) | 5.7 (4.5, 6.8) |
| 5-0 | 24 | 6.2 (5.4, 6.9) | 4.7 (4.0, 5.4) | 4.0 (3.1, 4.9) | 5.5 (5.1, 5.9) | 5.0 (4.2, 5.9) | 4.3 (3.5, 5.1) | 5.3 (4.2, 6.5) | 6.9 (6.4, 7.5) | 6.8 (6.1, 7.5) |
| 5-3 | 12 | 5.4 (4.3, 6.5) | 4.1 (3.1, 5.0) | 4.8 (3.3, 6.4) | 5.3 (4.5, 6.2) | 5.8 (5.0, 6.6) | 4.9 (4.0, 5.8) | 7.5 (6.9, 8.1) | 6.7 (6.0, 7.3) | 6.0 (4.8, 7.2) |
| 5-6 | 17 | 5.8 (4.7, 6.8) | 4.8 (3.8, 5.8) | 4.3 (3.2, 5.4) | 5.9 (5.9, 6.1) | 6.3 (5.6, 7.0) | 4.6 (3.8, 5.4) | 6.6 (5.3, 7.9) | 7.1 (6.5, 7.7) | 6.8 (5.9, 7.6) |
| 5-9 | 14 | 6.1 (5.2, 6.9) | 5.3 (4.2, 6.3) | 4.6 (3.5, 5.6) | 5.6 (5.2, 6.1) | 6.1 (5.2, 7.0) | 4.6 (3.8, 5.5) | 7.0 (5.7, 8.3) | 6.6 (6.1, 7.2) | 6.8 (6.0, 7.6) |
| 6-0 | 15 | 5.4 (4.4, 6.4) | 4.3 (3.2, 5.4) | 3.9 (2.9, 5.0) | 5.4 (4.4, 6.4) | 6.0 (5.3, 6.7) | 4.5 (3.2, 5.9) | 7.5 (7.0, 8.1) | 6.5 (5.4, 7.6) | 7.1 (6.3, 7.9) |
| 6-3 | 23 | 6.8 (6.1, 7.4) | 5.3 (4.3, 6.2) | 5.1 (4.0, 6.2) | 5.9 (5.6, 6.1) | 5.6 (4.7, 6.5) | 5.2 (4.7, 5.7) | 7.6 (7.0, 8.1) | 6.9 (6.3, 7.4) | 7.5 (7.0, 8.0) |
| 6-6 | 20 | 6.7 (5.9, 7.4) | 4.6 (3.4, 5.9) | 4.8 (3.9, 5.7) | 5.8 (5.6, 6.1) | 6.1 (5.4, 6.8) | 5.2 (4.4, 6.0) | 7.6 (7.0, 8.1) | 7.3 (6.9, 7.8) | 7.4 (6.8, 8.0) |
| 6-9 | 21 | 6.7 (6.0, 7.4) | 5.1 (4.2, 6.0) | 5.3 (4.5, 6.2) | 5.7 (5.1, 6.3) | 6.1 (5.4, 6.8) | 5.6 (5.2, 5.9) | 7.2 (6.3, 8.2) | 6.8 (6.2, 7.4) | 7.4 (6.9, 7.9) |
| 7-0 | 21 | 6.5 (5.6, 7.4) | 5.4 (4.7, 6.1) | 4.9 (4.1, 5.7) | 5.7 (5.1, 6.3) | 6.5 (5.8, 7.3) | 5.5 (5.0, 6.0) | 7.1 (6.3, 8.0) | 7.1 (6.5, 7.7) | 7.3 (6.8, 7.9) |
| 7-3 | 25 | 6.5 (5.8, 7.3) | 5.8 (5.3, 6.3) | 5.7 (4.8, 6.5) | 5.7 (5.9, 6.0) | 6.5 (5.9, 7.1) | 5.5 (5.2, 5.9) | 7.9 (7.7, 8.1) | 7.0 (6.5, 7.4) | 7.7 (7.4, 8.0) |
| 7-6 | 31 | 6.2 (5.5, 6.8) | 6.2 (5.6, 6.7) | 6.2 (5.5, 6.9) | 6.0 (6.0, 6.0) | 6.8 (6.4, 7.3) | 5.6 (5.2, 6.0) | 7.7 (7.3, 8.1) | 7.2 (6.9, 7.6) | 7.4 (7.0, 7.7) |
| 7-9 | 4 | 6.8 (5.2, 8.3) | 5.8 (3.7, 7.8) | 6.5 (3.5, 9.5) | 5.4 (4.4, 6.4) | 5.0 (0.9, 9.1) | 6.0 (6.0, 6.0) | 8.0 (8.0, 8.0) | 7.5 (5.9, 9.1) | 8.0 (8.0, 8.0) |

All values are reported as means (95 % confidence intervals). The presented scores are means of sum of two trials per FMS item for children in the associated age-ranges. Maximum score 4 criteria: 8; maximum score 3 criteria: 6. Age equivalents reported in three month intervals according to the TGMD-3 protocol, e.g.: 3-0 = 3 years 0 months up to 3 years 3 months; 3-3 = 3 years and 3 months up to 3 years and 6 months; 3-6= 3 years 6 months up to 3 years 9 months; 3-9= 3 years 9 months up to 4 years. *For the item “kick stationary ball” the original criteria number 2 (*child takes an elongated stride or leap just prior to ball contact*) was not evaluated, i.e. the score is based on the three remaining criteria (Ulrich et al., 2019); For the item “walk on line backward” the original criteria number B43 (*steps on line precisely without trial*) was not evaluated, i.e. the score is based on the four remaining criteria (Sun et al., 2010).
